# Controversy

**Published:** 1856-07

**Authors:** 


*Controversy.*

Our paragraph in the May number of this *Journal*, in relation to one in the Buffalo *Journal*, has called out three more pages from Dr. Palmer, in the columns of the *Peninsular Journal* for June. It is a re-hash of his former personal accusations, diluted to about the *fifth* Homœpathic *attenuation.*

The only remark we have to make on the subject at present, is, that any editor who repeatedly publishes to his readers the assertions, that his neighbor has used in controversy "*false* and *abusive* language"—has made "*untruthful* statements, *false* accusations, and *bitter bursts* of feeling"—has passed "to the extreme of *rude discourtesy* and *reckless mis-statements*"—and has been "his (the editor's) unjust accuser, nay his *bitter traducer:*" and yet, when called upon to do so, entirely refuses to place before his readers the identical language which he thus styles "false and abusive"—"untruthful"—"reckless," and *bitterly traducing*, is destitute of those ordinary principles of honor which should control the intercourse between intelligent men, and is, consequently, unworthy of any notice whatever.

